# The postnatal window is critical for the development of sex-specific metabolic and gut microbiota outcomes in offspring

**DOI:** 10.1080/19490976.2021.2004070

**Published:** 2021-11-23

**Authors:** Laurence Daoust, Béatrice S.-Y. Choi, Sébastien Lacroix, Vanessa Rodrigues Vilela, Thibault Vincent Varin, Stéphanie Dudonné, Geneviève Pilon, Denis Roy, Emile Levy, Yves Desjardins, Benoit Chassaing, André Marette

**Affiliations:** a Quebec Heart and Lung Institute Research Center, Quebec, Montreal, Canada; b Institute of Nutrition and Functional Food, Laval University, Quebec, Montreal, Canada; c Canada Research Excellence Chair in the Microbiome-Endocannabinoïdome Mediators Axis in Metabolic Health (Cerc-mend), Laval University, Quebec, Montreal, Canada; d Chu Sainte-Justine Research Center, Montreal University, Montreal, Canada; e Inserm U1016, Team “Mucosal Microbiota in Chronic Inflammatory Diseases”, Cnrs Umr 8104, Université De Paris, Paris, France

**Keywords:** Cross-fostering, postnatal environment, obesity, offspring, dams, polyphenols, cranberry, gut microbiota

## Abstract

The Developmental Origins of Health and Disease (DOHaD) concept has been proposed to explain the influence of environmental conditions during critical developmental stages on the risk of diseases in adulthood. The aim of this study was to compare the impact of the prenatal vs. postnatal environment on the gut microbiota in dams during the preconception, gestation and lactation periods and their consequences on metabolic outcomes in offspring. Here we used the cross-fostering technique, e.g. the exchange of pups following birth to a foster dam, to decipher the metabolic effects of the intrauterine versus postnatal environmental exposures to a polyphenol-rich cranberry extract (CE). CE administration to high-fat high-sucrose (HFHS)-fed dams improved glucose homeostasis and reduced liver steatosis in association with a shift in the maternal gut microbiota composition. Unexpectedly, we observed that the postnatal environment contributed to metabolic outcomes in female offspring, as revealed by adverse effects on adiposity and glucose metabolism, while no effect was observed in male offspring. In addition to the strong sexual dimorphism, we found a significant influence of the nursing mother on the community structure of the gut microbiota based on α-diversity and β-diversity indices in offspring. Gut microbiota transplantation (GMT) experiments partly reproduced the observed phenotype in female offspring. Our data support the concept that the postnatal environment represents a critical window to influence future sex-dependent metabolic outcomes in offspring that are causally but partly linked with gut microbiome alterations.

## Introduction

The Developmental Origins of Health and Disease (DOHaD) hypothesis refers to the influence of environmental factors during both the *in utero* and the postnatal developmental stages on the risk of developing chronic disease later in life.^1^This hypothesis has been put forward to explain the present disease burden our society is facing from an evolutionary perspective.^2^ Obesity, defined as an excessive fat accumulation, is a global epidemic that increases the risk of many comorbidities such as type 2 diabetes and cardiovascular diseases.^1,3^ Obesity is not only prevalent in adulthood but also affects an increasing proportion of children worldwide.^4^ Correlations between maternal dietary patterns and the health of offspring were first observed in a study conducted on rats in the 1970s by Zamenhof *et al*.^5^ Since then, considerable evidence supports the important role of the intrauterine environment in the programming of the development of obesity and diabetes later in life. Much of this evidence was gained from observational studies in humans based on the association between pre-pregnancy maternal BMI, gestational weight gain and neonatal adiposity.^6–8^ In animal models, both maternal undernutrition and overnutrition are associated with impaired glucose homeostasis and increased adiposity in offspring, an effect that persists until adulthood.^9–12^ Also, mounting evidence indicates that the gut microbiota is a key player in obesity development, low-grade chronic inflammation and dysmetabolism.^13,14^ The maternal gut microbiota during pregnancy has recently been proposed as a determinant of neonatal metabolic health.^15^ On the other hand, it has been suggested that the postnatal environment could override some of the prenatal and genetic factors involved in programming the development of metabolic outcomes in the offspring.^16^ Indeed, it is known that the first days of life are critical in the development of the immune system of newborns. Colonization of the gut by bacteria from the environment plays an important role in programming the maturation of the gastrointestinal tract^17,18^ and is influenced by the nursing dam, pointing toward the postnatal environment as an important factor in shaping future metabolic outcome.^19^ However, the respective contributions of *in utero* versus postnatal events on the development of metabolic diseases in offspring needs to be clarified. The use of the cross-fostering (CF) technique, which has been defined by Daft *et al*.^19^ as “the switching of newly born pups to non-birth dams who themselves have recently had pups or are ready to nurse”,^19(p.2)^ can therefore allow deciphering the metabolic effects of intrauterine versus postnatal environmental exposures.

Manipulation of the gut microbiota by the administration of prebiotics has been put forward as a promising target to alleviate metabolic diseases. Multiple studies have highlighted the beneficial effects of consuming polyphenol-rich fruits or their extracts in the prevention of obesity-associated metabolic disturbances.^20^ We have previously shown that a polyphenol-rich cranberry extract (CE) prevents the development of metabolic diseases in male mice following diet-induced obesity in association with a major shift in the composition of the gut microbiota.^21,22^ The aim of this study was to evaluate using a cross-fostering approach the contribution of the prenatal vs. postnatal environment to CE polyphenols on the offspring submitted to an obesogenic environment. Despite an improved metabolic profile in CE-treated dams, female but not male offspring nursed by these dams presented a deleterious metabolic phenotype.

## Methods/materials

### Cranberry extract characterization

The CE (CRA-std-Pur Lot #252) was produced by Nutra-Canada, now Diana Food Canada (Québec, Canada). The extract was diluted in water from the animal facility at a concentration of 40 mg/ml. The extract contains 34.2% of polyphenols including 10.7% of proanthocyanidins (PACs) (**Supplementary Table 1**). The methodology to characterize polyphenols present in the extract has been described previously.^22^

### Dam’s treatment

All animal experiments were approved by Laval University Animal Ethics Committee (2016087-1). Six-week-old C57BL/6J female mice (n = 21; Jackson Laboratories, Sacramento, USA) were housed one per cage on a chow diet (Teklad Global 18% Protein Rodent Diet, Envigo) in a controlled environment (12 h daylight cycle, lights off at 18:00) with food and water *ad libitum* in the animal facility of the Quebec Heart and Lung Institute (Quebec, Canada). After 2 weeks of acclimatization, mice were fed a high-fat/high-sucrose (HFHS) diet (Research diet D16120503) containing 56% kcal from fat, 27% kcal from carbohydrates and 17% kcal from proteins. They were gavaged daily with either 200 mg/kg of a cranberry polyphenol-rich CE (n = 7), as previously used^21,22^ or with the equivalent volume of water as the control (Veh-treated) (n = 14) for a period of 14–19 weeks, i.e.until they were euthanized, which is referred to as the treatment period (Figure 2a). This period has been chosen to allow a minimum of 8 weeks CE-treatment in dams before birth to induce obesity-associated metabolic improvement as previously published.^22^

### Reproduction

Between weeks 8 and 12 of treatment, Veh-treated and CE-treated female mice were mated with C57BL/6J male mice fed a standard chow diet (Figure 2a). Each female mouse was paired with a male for a period of 16 h overnight and they were both fed HFHS diet during this period. After mating, pregnancy was determined by assessing the presence of a vaginal plug, which was considered as d 0 of the pregnancy. If no plug was observed, female mice underwent the same mating protocol again. If after seven consecutive matings the female mice were still not pregnant, they were euthanized. During pregnancy, mice were closely monitored and gavaged daily with the CE or with water as the vehicle until the day of birth. One week prior to the offspring’s birth, dams were fasted for 6 h. A blood sample was collected by the saphenous vein and the glycemia was taken using an Accu-Check glucometer (Bayer).

### Cross-fostering

**Figure 1.**
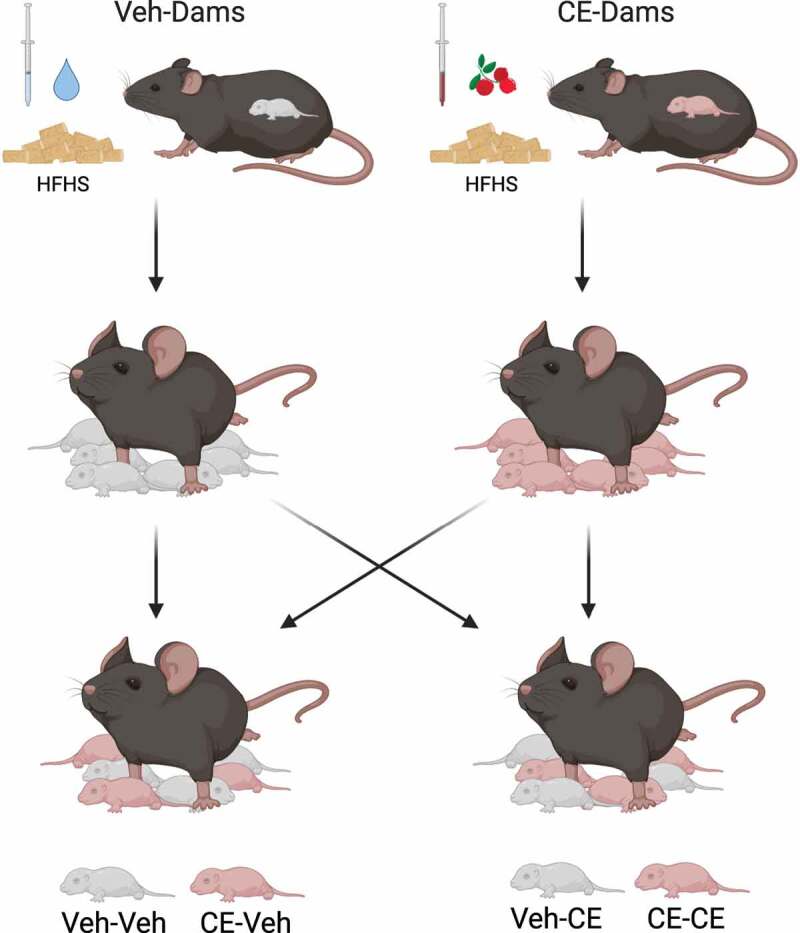
Schematic representation of cross-fostering. Dams were fed an HFHS diet and daily gavage with the Veh or the CE for a period of 14–19 weeks. Dams were mated and in an approximate range of 24 h following birth of offspring, about half of their litter was cross-fostered, that is, from a Veh-dam to a CE-dam and from a CE-dam to a Veh-dam. Offspring were then weaned by their adoptive mothers. Mice in the second half of the litter were weaned by their biological mothers. The figure was created with BioRender.com and a valid subscription that allows publication of the created content

Dams gave birth after 11–15 weeks of treatment. Within approximately 24 h following birth, i.e., on the day where the newborn offspring were found considering that we would assess birth every morning during gestation, dams were removed from their cage for the time required to proceed to the exchange of pups (approximately 10 min). The sex of the offspring was determined by visually assessing the genital-anal length. Both male and female offspring were kept. Approximately half of the litter stayed with their biological dams, while the other half of the litter was exchanged (Figure 1). Offspring were randomly picked based on gender and coated, with the litter of their adoptive dam to increase their chance of acceptance into the new litter. Adoptive offspring were identified with a tattoo under their foot before the exchange to differentiate them from the biologically nursed offspring. Offspring that did not survive the first 12 h of life or who were presenting abnormalities at birth were removed from the litter and euthanized in accordance with the ethical guidelines. The day of the CF was considered as the first day of the nursing period. After being nursed for 21 d, dams were removed from the cage and euthanized between 14 and 19 weeks of treatment by cardiac puncture under general anesthesia with isoflurane. Organs and tissues were collected and snap frozen in liquid nitrogen. Blood was drawn and centrifuged to separate the plasma. Organs and plasma were stored at −80°C.

**Figure 2.**
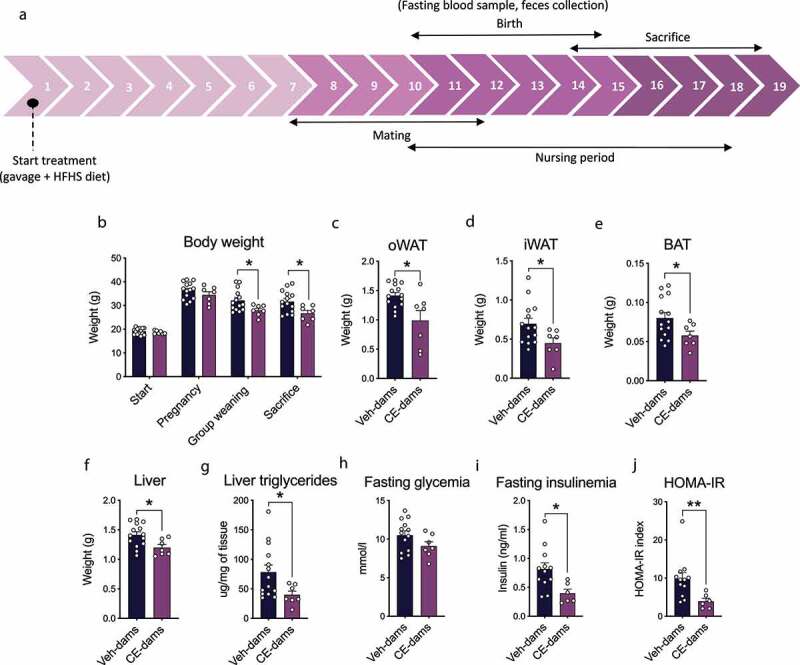
Impact of the administration of a polyphenol-rich CE in dams on metabolic parameters. (a) Timeline of experiments displayed in weeks. (b) Body weight at different time points, (c) ovarian white adipose tissue (oWAT), (d) inguinal white adipose tissue (iWAT), (e) brown adipose tissue (BAT), (f) liver weight and (g) liver triglycerides. Mice were fasted for 6 h before assessing (h) fasting glycemia, (i) fasting insulinemia and (j) HOMA-IR index. A Student t-test (for parametric data sets) or Mann–Whitney test (for non-parametric data sets) were used to assign significance to the differences between groups. Differences between groups at different time points were assessed using a two-way repeated measures ANOVA with a Holm–Sidak *post hoc* test. Values are expressed as the mean ± SEM (Veh-dams n = 14; CE-dams n = 7). * *P* < .05; **P < .01

### Offspring treatment

After 21 d, offspring were separated from the dams. They stayed in the same cage as their siblings for an additional period of 3 d, i.e., until the 24th day to facilitate weaning. After this period, offspring were single caged which is considered as time zero (T0) and fed the same HFHS diet as the dams for 8 weeks (Figure 4a). Offspring born from a dam receiving the vehicle and nursed by this same dam were identified as Vehicle–Vehicle (Veh-Veh; male n = 16; female n = 17). Offspring born from a dam receiving the CE and nursed by this same dam were identified as Cranberry–Cranberry (CE-CE; male n = 8; female n = 9). Offspring born from a control dam but adopted by a dam receiving the CE were identified as Vehicle–Cranberry (Veh-CE; male n = 8; female n = 9). Finally, offspring born from a dam receiving the CE treatment but adopted by a control dam were identified as Cranberry–Vehicle (CE-Veh; male n = 17; female n = 9) (Figure 1). Body weight and food intake were measured three times a week. Body composition analysis was performed at T0 and after 8 weeks of treatment by quantitative nuclear magnetic resonance (qNMR). After 8 weeks of HFHS treatment (11 weeks of age), offspring were fasted overnight for a period of 12 h and an oral glucose tolerance test (OGTT) was performed. Blood glucose concentration was measured before (Time 0) and 15, 30, 60, 90 and 120 min after the administration of a 50% dextrose solution (2 μl/g). Blood was collected at every time point during the OGTT and centrifuged (3500 rpm, 10 min at 4°C). The plasma was then collected and stored at −80°C. Insulinemia was determined by ELISA (Alpco Mouse Ultrasensitive Insulin kit) according to the manufacturer’s instructions. Offspring were euthanized by the same technique described previously for dams. Organs and tissues were collected and snap frozen in liquid nitrogen. Blood was drawn and centrifuged to separate the plasma. Organs and plasma were stored at −80°C.

### Gut microbiota transplantation (GMT)

GMT solutions were prepared from the cecum content of every female offspring collected at euthanasia (T8) as metabolic impairments in female offspring donor mice were observed at this time point. Cecum contents from each group were combined and diluted in cold sterile PBS (20 µl/mg of feces), vortexed for 15 min at maximum speed, centrifuged for 5 min at 4 °C at 300 rpm and then aliquoted. Each aliquot was thawed only once, when gavaged. Each mouse received 150 μl of their respective GMT solution twice during the first two days of the study and once every week for the subsequent 3 weeks.

For this study, 3-week-old C57BL/6J female mice (n = 31 (7–8 mice/group); Jackson Laboratories, Sacramento, USA) were housed one per cage on a chow diet (Teklad Global 18% Protein Rodent Diet, Envigo) in a controlled environment (12 h daylight cycle, lights off at 18:00) with food and water *ad libitum* in the animal facility of the Quebec Heart and Lung Institute (Quebec, Canada). After 4 d of acclimatization, mice were given antibiotics (ampicillin (1 g/L), neomycin (0,5 g/L)) in their drinking water for the subsequent 7 d to ensure depletion of the intestinal microbiota (Figure 9a). On d 8 (T0), water containing antibiotics was removed and mice were housed in sterile cages, given sterile water and fed an HFHS diet (Research diet Irradiated D16120503I) containing 56% kcal from fat, 27% kcal from carbohydratesand 17% kcal from proteins. Fresh feces were also collected. From this point and throughout the entire study, mice were only manipulated under a level 2 biosafety cabinet using sterile gloves. On d9, mice were fasted for 2 h and gavage five times every 30 min with 75 μl of a sterile polyethylene glycol (PEG) solution as previously described^23^ to ensure complete emptying of the intestinal content and avoid residual antibiotics to interfere with GMT. First GMT was performed on this same day, 6 h after the last gavage of PEG solution. A second GMT was performed on d10 and once every week afterward to maintain bacterial colonization and avoid environmental pressure. Food intake and body weight was measured three times a week. Body composition analysis was performed at T0 and after 4 weeks of treatment by qNMR. After 4 weeks of treatment (8 weeks of age), GMT-receiving female mice were fasted overnight for a period of 12 h and an oral glucose tolerance test (OGTT) was performed. Blood glucose concentration was measured before (Time 0) and 15, 30, 60 and 120 min after the administration of a 50% dextrose solution (2 μl/g). Blood was collected at every time point during the OGTT and centrifuged (3500 rpm, 10 min at 4°C). The plasma was then collected and stored at −80°C. Insulinemia was determined as mentioned above. During the fifth week after the first GMT, female mice were euthanized by the same technique described above for dams. Organs and tissues were collected and snap frozen in liquid nitrogen. Blood was drawn and centrifuged to separate the plasma. Organs and plasma were stored at −80°C.

### Determination of hepatic triglycerides

Hepatic triglycerides were extracted using the chloroform-methanol Folch lipid extraction method as previously described.^22^ Quantification of liver triglycerides was assessed with an enzymatic reaction from a commercial kit (Infinity Triglycerides Reagent, Thermo Fisher Scientific) according to the manufacturer’s instructions.

### Laser capture microdissection and bacterial DNA extraction from colon samples

Freshly dissected colon samples were fixed in a water-free Carnoy fixative solution (60% methanol, 30% chloroform, 10% glacial acetic acid) to ensure preservation of the colonic mucus layer. Tissues were embedded in paraffin with a vertical orientation. Then, 5 μm sections were obtained and transferred on PEN Membrane Frame Slides (Thermo Fisher Scientific). Once deparaffinized, samples were immunostained as previously described.^24^ Samples were microdissected using the Arcturus*XT*TM Microdissection Systems (Applied Biosystems). Inner mucus-associated bacterial DNA was extracted from laser capture microdissected colon samples with the QIAamp® DNA Micro Kit (Qiagen) according to the manufacturer’s instructions (n = 3–6/group).

### 16S rRNA high-throughput sequencing and analysis of fecal and mucus sequencing data

Fresh feces were harvested and snap frozen at −80°C from gestating dams 3–5 d before delivering their offspring and from offspring at T0 and T8. Fresh feces Fecal DNA was extracted with the ZymoBIOMICS DNA Miniprep kit (Zymo Research) according to the manufacturer’s instructions (n = 6/offspring group). Offspring for whom the body weight gain throughout the 8 weeks of HFHS treatment was the closest to the average in their respective group were first chosen for 16S rRNA high-throughput sequencing. From these chosen mice, minor changes were made in order to avoid significant outliers for glucose homeostasis parameters. For each DNA sample, amplification of the V3-V4 region was performed using the primers 341 F (5-CCTACGGGNGGCWGCAG-3) and 805 R (5-GACTACHVGGGTATCTAATCC-3) (Illumina, CA, USA). Libraries were purified using AxyPrep magnetic beads (Axygen Biosciences, CA, USA), and libraries were assessed using DNA 7500 chips (Agilent Technologies, CA, USA) and picogreen (Life Technologies, CA, USA). High-throughput sequencing (2 × 300 bp paired-end) was performed on a MiSeq platform (Illumina, CA, USA) at the IBIS (Institut de Biologie Intégrative et des Systèmes, Université Laval). Sequences were processed using the DADA2 package (v1.10.1)^25^ in the R environment (http://www.R-project.org), and associations to bacterial taxa were obtained using the RDP classifier algorithm (v2.2)^26^ trained against the Silva database 132.^27^ The functional profile of KEGG Orthology (KO) for each sample was predicted from 16S rRNA amplicon data with Tax4Fun.^28^ In order to normalize sampling effort, samples were rarefied to an even sampling depth of 14,198 and 10,212 sequences for fecal and mucus sequencing data, respectively.

### Statistical analysis

A Student t-test (for parametric data sets) or Mann–Whitney test (for non-parametric data sets) were used to assign significance to the differences between Veh-dams and CE-dams (GraphPad Prism 8, USA). A Shapiro–Wilk normality test was applied to determine whether data would be treated as parametric or non-parametric. The significance between offspring groups was assessed using a two-way ANOVA with a Holm–Sidak *post hoc* test. When assessing the effect at different time points, a two-way repeated measures ANOVA with a Holm–Sidak *post hoc* test was used (SigmaPlot 12 Software, USA). In order to respect ANOVA postulates, logarithmic transformations were carried out when necessary. Principal coordinates analysis (PCoA) plots were produced using Bray–Curtis dissimilarity index.^29^ In order to assign statistical significance to the differences between clusters of sample bacterial profiles, preliminary tests of homogeneity of variances among groups followed by Permutational Multivariate Analysis of Variance (PERMANOVA) tests were carried out using the “Adonis” function of the R package “vegan” (v2.5.4), with 1000 permutations and subsequent Bonferroni–Holm P adjustment. We applied the linear discriminant analysis effect size (LEfSe) method for differential abundance analysis for fecal and mucus sequencing data.^30^ A linear discriminant analysis (LDA) score ≥ 2.5 was considered statistically significant. Spearman’s rank correlation was used to assess the degree of association between metabolic parameters and bacterial taxa. Data are expressed as mean ± SEM. All results were considered statistically significant at *p* < .05.

## Results

### CE treatment improves features of the metabolic syndrome in preconception, gestating and lactating dams

At the beginning of the treatment (start) and the end of the pregnancy (pregnancy), the body weight of dams was similar between groups, while CE-dams had a significantly lower body weight at the time where offspring were separated from them (group weaning) and at the end of the treatment (sacrifice) when compared to the Veh-dams (Figure 2b). The weights of the inguinal white adipose tissue (iWAT), the intrascapular brown adipose tissue (BAT) and the ovarian white adipose tissue (oWAT) were significantly reduced in CE-dams (Figure 2c–e). Liver weight (figure 2f) and liver triglycerides (Figure 2g) were also significantly reduced in CE-dams compared to the Veh-dams. While fasting glycemia was slightly but not significantly reduced in CE-dams (Figure 2h), we found that both fasting insulinemia (Figure 2i) and the HOMA-IR insulin resistance index (Figure 2j) to be significantly decreased, indicating an improvement of insulin sensitivity in CE-dams. Thus, our data demonstrate that the polyphenol-rich CE treatment improved the metabolic profile in dams in association with a lower body weight gain.

### CE treatment induces specific changes in the fecal microbiota of dams but is not associated with change in the mucus-associated microbiota

**Figure 3.**
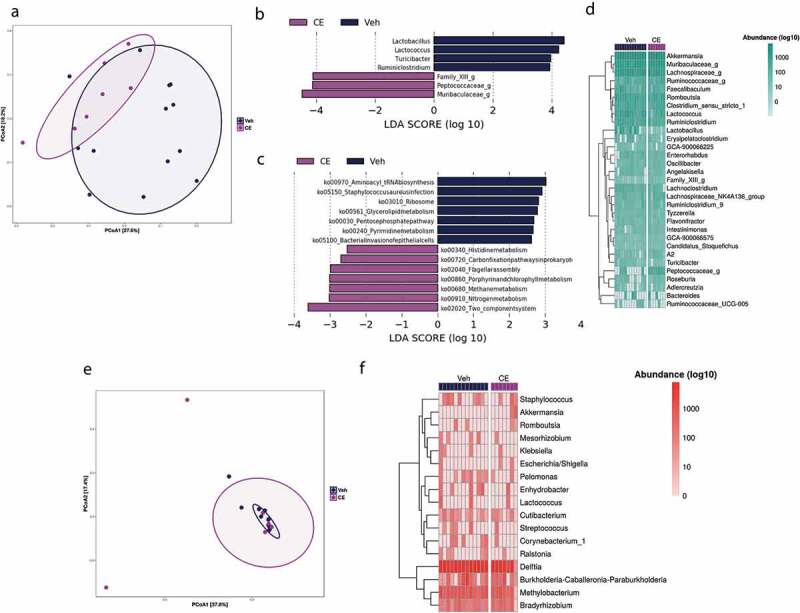
Impact of the administration of a polyphenol-rich CE on the gut microbiota composition and the inner-mucus associated bacterial profile in dams. (a) β-diversity of the gut microbiota among groups was observed by means of PCoA using on Bray–Curtis dissimilarity index. (b, c) LEfSe was calculated in order to assess the taxa and functions, respectively, that more strongly discriminates the composition of the gut microbiota of Veh-dams from CE-dams. (d) Heat map represents the abundance of taxa (log10 transformed) detected in the gut microbiota of dams (Veh-dams n = 14; CE-dams n = 7). (e) β-diversity of the inner-mucus associated bacteria was observed between Veh-dams and CE-dams using means of PCoA using on Bray–Curtis dissimilarity index. (f) Heat map represents the abundance of taxa (log10 transformed) detected in the inner-mucus associated microbiota of dams (Veh-dams n = 13; CE-dams n = 7)

Fecal samples were collected from gestating dams 3–5 d before delivering their offspring. PCoA of the 16S rRNA gene amplicon sequences revealed that the fecal microbiome of CE-treated dams cluster partially, but significantly (*P* < .05; R^2^ = 0.11) apart from the vehicle-treated mice (Figure 3a). LEfSe was used to determine genera that differed between Veh-dams and CE-dams. This analysis revealed that the gut community of CE-dams was distinguishable from the Veh-dams by the increased relative abundance of *Muribaculaceae, Peptococcaceae, Family XIII* as well as the decreased abundance of *Lactobacillus, Lactoccoccus, Turicibacter* and *Ruminiclostridium* (Figure 3b). The use of KEGG Orthology to predict the functional alterations in the gut microbiota of dams revealed an increase in functional pathways associated with energy metabolism (carbon fixation pathway in prokaryotes, methane metabolism, nitrogen metabolism) as well as cofactor and vitamins metabolism (porphyrin and chlorophyll metabolism) in CE-dams. Increased amino acid metabolism (histidine metabolism), signal transduction (two-component system) and cell motility (flagella assembly) functions were also overrepresented in the gut microbiota of CE-dams. Conversely, increased lipid metabolism (glycerolipid metabolism), carbohydrate metabolism (pentose phosphate pathway), nucleotide metabolism (pyrimidine metabolism), infectious disease (staphylococcus aureus infection), translation (aminoacyl tRNA biosynthesis, ribosomes), as well as glycan biosynthesis and metabolism (bacterial invasion of epithelial cells) functional pathways were overrepresented in Veh-dams (Figure 3c). The relative abundance of bacterial taxa detected in fecal samples of Veh-dams and CE-dams is represented in Figure 3d.

**Figure 4.**
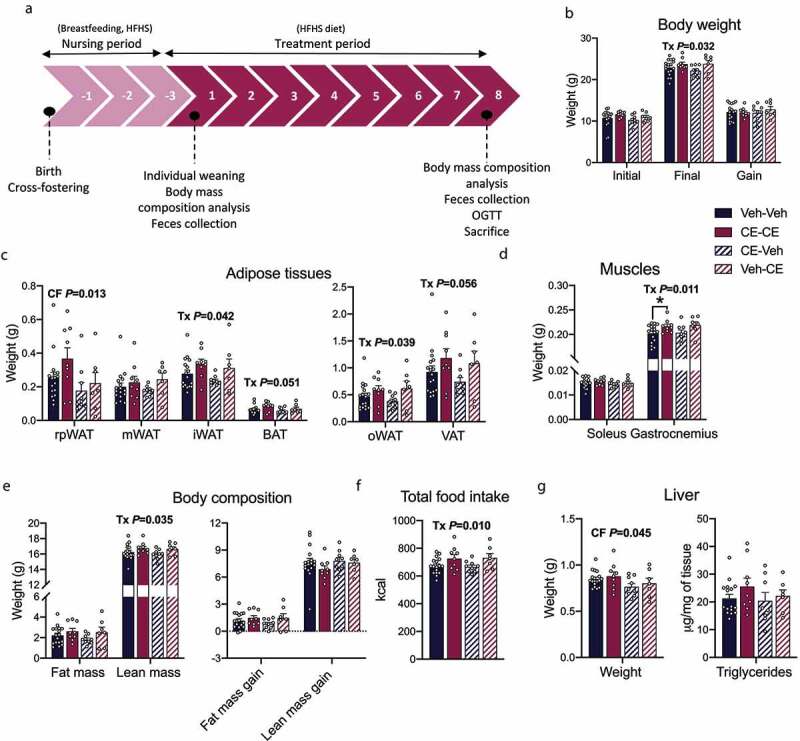
Effect of the postnatal environment on metabolic parameters in female offspring. (a) Timeline of experiments displayed in weeks. (b) Initial, final, and body weight gain. (c) Weight of different adipose tissues. (d) Weight of the soleus and the gastrocnemius. (e) Fat and lean mass and gain of fat and lean mass . (f) Total food intake. (g) Liver weight and liver triglycerides. The effect of the cross-fostering on offspring’s biological parameters is referred to as “CF.” The effect of the treatment of the nursing mother (CE vs. Veh-dams) on offspring’s biological parameters is referred to as “Tx.” Two-way ANOVA analysis with a Holm–Sidak *post hoc* test was used to evaluate the difference between groups. rpWAT: retroperitoneal adipose tissue; oWAT: ovarien adipose tissue; mWAT: mesenteric adipose tissue; iWAT: inguinal white adipose tissue; BAT: brown adipose tissue; VAT: visceral adipose tissue. Data are expressed as the mean ± SEM (Veh-Veh n = 17; CE-CE n = 9; CE-Veh n = 9; Veh-CE n = 7)

Since selective changes in mucosa-associated microbiota can also inform on more specific microbial disturbances in chronic inflammatory intestinal disorders,^31,32^ we next explored potential alterations in bacterial genera of laser-captured microdissected inner mucus layer by 16S rRNA gene amplicon sequencing.^24^ PCoA analysis of these data showed no significant separation (*P* > .1) of CE-dams vs. Veh-dams (Figure 3e). Whereas the inner mucus microbiota of dams revealed a wide range of mucus-specific taxa, we did not observe any differences between CE-dams vs. Veh-dams mucosa samples (figure 3f).

### Postnatal CE-treatment in dams exacerbates weight gain and glucose intolerance in HFHS-fed female offspring

At the time of weaning, all offspring were placed on a HFHS diet for 8 weeks. Multiple metabolic parameters were evaluated in both male and female to assess the effect of the dam’s CE vs. Veh treatments on future metabolic outcomes in offspring (Figure 4a**; Supplementary Tables 2 and 3**). Male offspring did not show any changes in metabolic phenotypes between groups (**Supplementary Table 2**). Thus, even though the CE-dams were metabolically healthier, male offspring from both groups did develop metabolic disorders associated with obesity, and to the same extent as their counterparts nursed by a Veh-dam. In marked contrast, female offspring showed many changes in physiological parameters that were affected by CE treatment. Indeed, CE-CE and Veh-CE female offspring mouse groups that were both nursed by CE-dams showed a significant increase in the final body weight (*P* = .032) compared to Veh-dams-nursed pups (Figure 4b). This could be explained by a significant increase of the iWAT (*P* = .042), the oWAT (*P* = .039) (Figure 4c) and the gastrocnemius muscle (*P* = .011) (Figure 4d) in comparison with the female offspring nursed by Veh-dams (Veh-Veh, CE-Veh). The weight of the BAT (*P* = .051) and visceral adipose tissues (*P* = .056) were borderline significant in CE-CE and Veh-CE female offspring (Figure 4c). In addition, lean mass was also significantly increased in female offspring nursed by CE-dams (CE-CE, Veh-CE) (*P* = .035) (Figure 4e). The increased total energy intake that was observed in Veh-CE and CE-CE female offspring (*P* = .010) (figure 4f) might have contributed to the increased accretion of adipose tissue depots in these animals. The adoption of offspring by either Veh or CE-dams had an impact on the decreased weight of the retroperitoneal white adipose tissue weight (*P* = .013) (Figure 4c) and of the liver (*P* = .045) (Figure 4g) in Veh-CE and CE-Veh female offspring. Overall, these results suggest that the nursing of female, but not male, offspring by a CE-treated dams aggravates the metabolic programming associated with an obesogenic diet during postnatal development.

**Figure 5.**
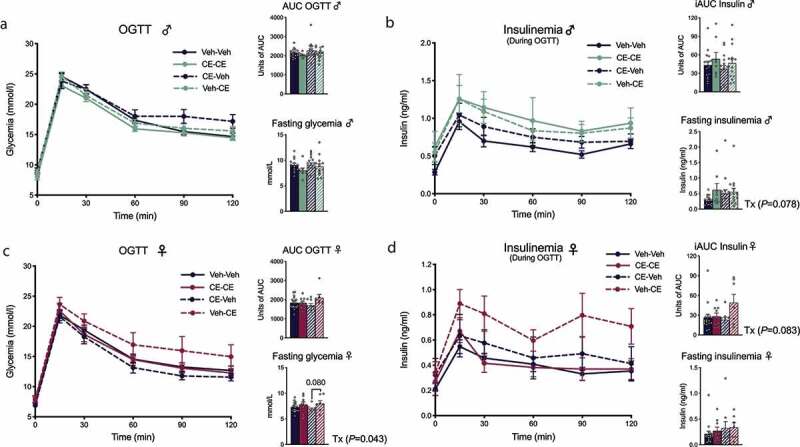
Effect of the postnatal environment on glucose homeostasis in male and female offspring. (a, b) Male and (c, d) female offspring were fasted for 12 h and an OGTT was performed (a–c). Insulinemia during the OGTT was measured by ELISA (b–d). Two-way ANOVA with a Holm–Sidak *post hoc* test was used to assess the significance of the differences between groups. Two-way ANOVA with repeated measures with a Holm–Sidak *post hoc* test was used to assess differences between groups at different time points. Data are expressed as the mean ± SEM (male offspring: Veh-Veh n = 16; CE-CE n = 8; CE-Veh n = 17; Veh-CE n = 15, female offspring: Veh-Veh n = 17; CE-Veh n = 9; CE-CE n = 9; Veh-CE n = 7)

We next performed an OGTT after 8 weeks of treatment in male and female offspring. In the male offspring, the OGTT did not reveal any significant difference in glucose management (Figure 5a,b). However, male offspring nursed by CE-dam (Veh-CE and CE-CE) remained the groups with the highest insulinemia during the test (Figure 5b). The same pattern was observed when measuring fasting insulinemia (*P* = .078) (Figure 5b). As compared to vehicle nursed females, CE-CE and Veh-CE female offspring groups nursed by CE-dams had a significantly increased fasting glycemia (*P* = .043) (Figure 5c). However, the treatment did not have any effect on blood glucose levels after the administration of the glucose bolus at different time points (Figure 5c). The assessment of insulinemia during the OGTT did not reveal any difference between treated groups at any point of the curve, although the AUC tended to be higher in Veh-CE and CE-CE female offspring (*P* = .083) (Figure 5d). Our results are in accordance with the general metabolic phenotype reported in Figure 4 and suggest that female offspring nursed by a CE-dam have an altered metabolic phenotype when fed a HFHS diet compared to their counterparts nursed by a Veh-dam. Again, these results suggest that the postnatal environment is an important determinant of future metabolic outcomes.

### The postnatal environment is key to determine the community structure of the gut microbiota

**Figure 6.**
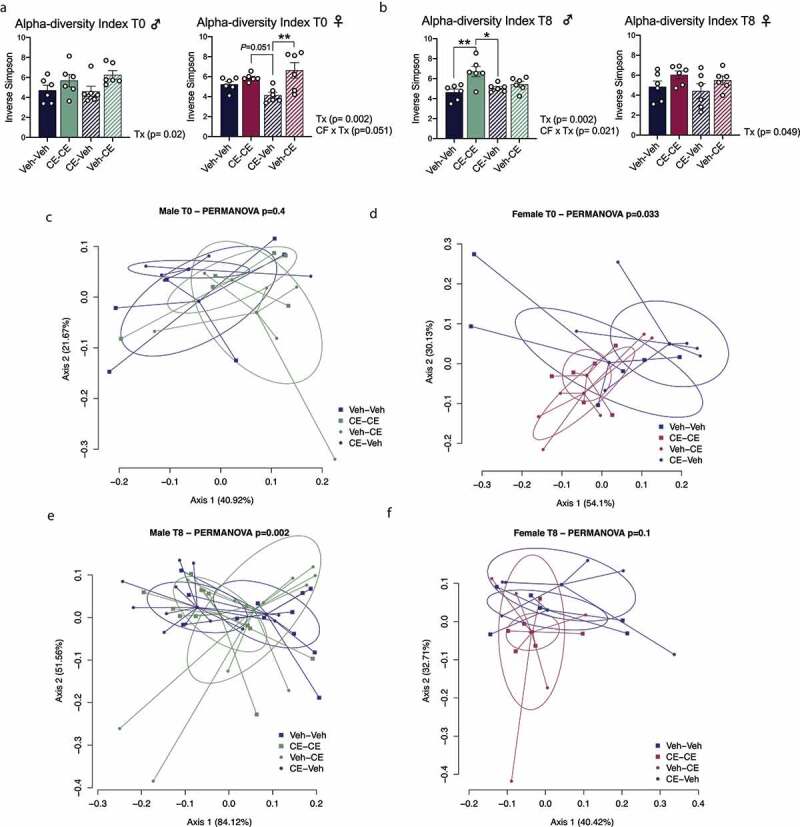
Fecal microbiota diversity indices in male and female offspring. (a, b) Inverse Simpson alpha-diversity index in male and female offspring at T0 (a) and T8 (b). (c,d) Plot of the PCoA of samples from male (c) and female offspring (d) at T0. (e, f) Plot of the PCoA of samples from male (e) and female (f) offspring at T8 using Bray–Curtis dissimilarity index. Ellipses on the PCoA represent 95% confidence interval. * *P* < .05; **P < .01 (n = 6/group)

We then analyzed the composition of the gut microbiota of male and female offspring at both T0 (at weaning) and T8 (after HFHS diet treatment). We observed significantly increased α-diversity in male and female offspring nursed by a CE-dam (CE-CE and Veh-CE) at T0 compared to the offspring nursed by a Veh-dam (CE-Veh and Veh-Veh) (Figure 6a). Interestingly, this increased α-diversity was maintained throughout the 8-week HFHS-feeding period in both male and female offspring (Figure 6b). When assessing β-diversity after weaning (T0), we did not observe any difference in male offspring (Figure 6c). However, at T8 when offspring were single-caged (*P* < .05; R^2^ = 0.29), the fecal microbiomes of male CE-CE vs. Veh-Veh groups (*P* = .004) and CE-Veh vs. CE-CE groups (*P* = .0015) significantly clustered apart (Figure 6e). On the other hand, female offspring showed significant differences in microbiome clusters at T0 (*P* < .05; R^2^ = 0.23). The microbiomes of female CE-CE vs. Veh-CE groups (*P* = .009), CE-Veh vs. CE-CE groups (*P* = .016) and CE-Veh vs. Veh-CE groups (*P* = .03) also significantly clustered apart (Figure 6d). No difference was seen at T8 in female offspring (figure 6f). Bray–Curtis distance was also calculated between offspring biological and adoptive dams at T0 and T8 (**Supplementary Figure 1**a,b). We observed lower distance from biological to adoptive dams in male offspring at T0 and T8, an effect that only reached significance at T8. Bray–Curtis distance was similar between biological and adoptive dams of female offspring at T0 and T8. A simultaneous timing of collection for both dams and offspring could have resulted in a more significant resemblance of the gut microbiome of fostered offspring to their adoptive mother than to their biological mother. Overall, these data suggest a stronger influence of the nursing mother than the biological mother on the establishment of the gut microbiota in offspring.

### Male and female offspring nursed by a CE-dam acquired distinct bacterial genera

**Figure 7.**
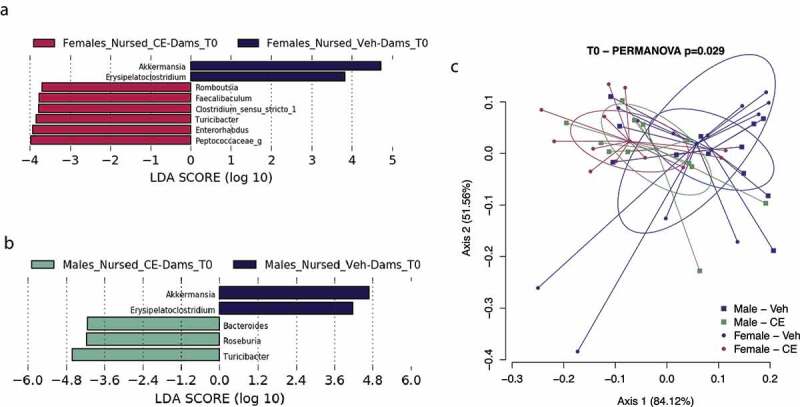
Effect of the postnatal environment on the development of the gut microbiota composition in male and female offspring. (a, b) LEfSe was calculated in order to assess the taxa that more strongly discriminates the composition of the gut microbiota in the postnatal environment at T0 in female (a) and male (b) offspring. (c) β-diversity of the fecal microbiome at T0 was observed between postnatal groups in male and female offspring using means of PCoA on Bray–Curtis dissimilarity index (n = 6/group). Ellipses on the PCoA represents 95% confidence interval

As shown in Figure 6, the postnatal environment mostly shapes the gut microbiota composition in offspring. Thus, this effect was assessed more specifically using LEfSe. Both male and female offspring nursed by a Veh-dam, that is, the Veh-Veh and the CE-Veh groups, were overrepresented at T0 with *Akkermansia* and *Erysipelatoclostridium* genera compared to their respective counterparts nursed by a CE-dam (Figure 7a,b). The microbiota of male offspring nursed by a CE-dam were overrepresented with *Turicibacter, Roseburia* and *Bacteroides* genera (Figure 7a), whereas the microbiota of female offspring nursed by a CE-dam were overrepresented with *Peptoccocaeae_g, Enterorhabdus, Turicibacter, Clostrodium_Sensu_Stricto_1, Faecalibacterium* and *Romboutsia* genera (Figure 7b). Those groups were also significantly different when assessing global community structure using β-diversity (*P* < .05; R^2^ = 0.12). Indeed, both female and male offspring of Veh-dams and CE-dam’s microbiomes clustered apart from each other at T0 (*P* = .085 and 0.056, respectively) (Figure 7c). Individual comparisons are presented in **Supplementary Figure 1**c-f. These results suggest that Veh-dams had a similar impact on the establishment of the gut microbiota in both male and female offspring during the postnatal period. Importantly, however, the gut microbiome of female offspring nursed by a CE-dam evolved differently than that of the male offspring also nursed by a CE-dam.

### Correlation between gut microbiota members and body composition, organ weight and glucose homeostasis parameters in male and female offspring

**Figure 8.**
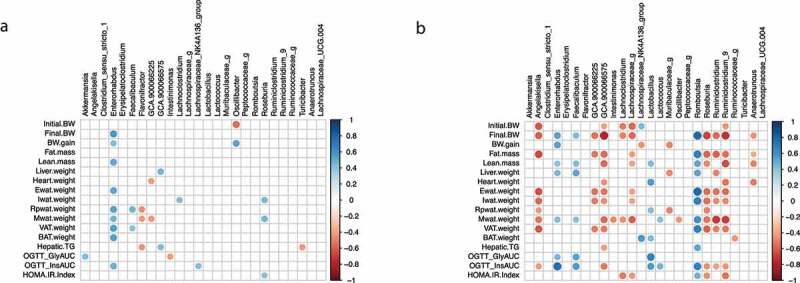
Correlation between metabolic parameters and the gut microbiome in male and female offspring. (a, b) Spearman’s rank correlation was used to assess the degree of association between metabolic parameters and 26 bacterial taxa that were present in more than 50% of all our samples in male (a) and female (b) offspring. Only significant associations are represented by the colored dots

We correlated different anthropometric and systemic parameters associated with body weight and glucose homeostasis with the 26 bacterial genera that were present in more than 50% of all our samples in male (Figure 8a) and female offspring (Figure 8b). Overall, female offspring showed a significantly stronger correlation than male offspring. For instance, *Angellakisella, Roseburia, Ruminiclostridium, Ruminiclostridium_9* genera and an unknown genus showed the strongest negative correlation with the body composition and organ weight. *Rombutsia, Enterorhabdus and Lactobacillus* showed the strongest positive correlation with parameters associated with body composition, organ weight and the AUC during the OGTT and insulinemia.

**Figure 9.**
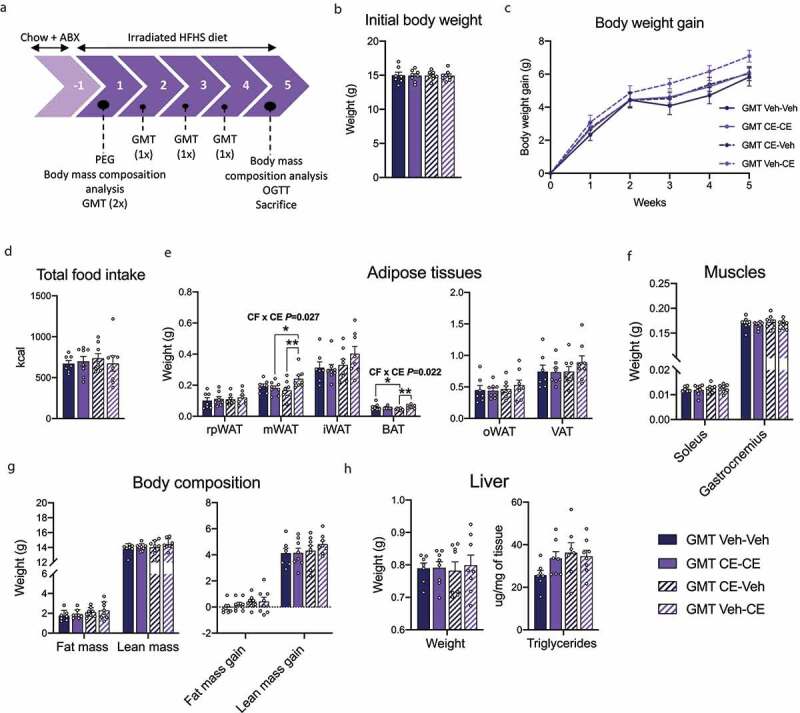
Impact of GMT on metabolic parameters associated with an HFHS diet in young female mice. (a) Timeline of experiments displayed in weeks. (b) Initial body weight. (c) Body weight gain. (d) Total food intake. (e) Weight of different adipose tissues. (f) Weight of the soleus and the gastrocnemius. (g) Fat and lean mass and gain of fat and lean mass . (h) Liver weight and triglycerides. Two-way ANOVA analysis with a Holm–Sidak *post hoc* test was used to evaluate the difference between groups. Two-way ANOVA with repeated measures with a Holm–Sidak *post hoc* test was used to assess differences between groups at different time points. ABX: antibiotics; rpWAT: retroperitoneal adipose tissue; oWAT: ovarien adipose tissue; mWAT: mesenteric adipose tissue; iWAT: inguinal white adipose tissue; BAT: brown adipose tissue; VAT: visceral adipose tissue. Data are expressed as the mean ± SEM (n = 7–8/group). * *P* < .05; **P < .01

### The gut microbiota is causally implicated in the exacerbated phenotype associated with an HFHS diet in CE-CE and Veh-CE female offspring

**Figure 10.**
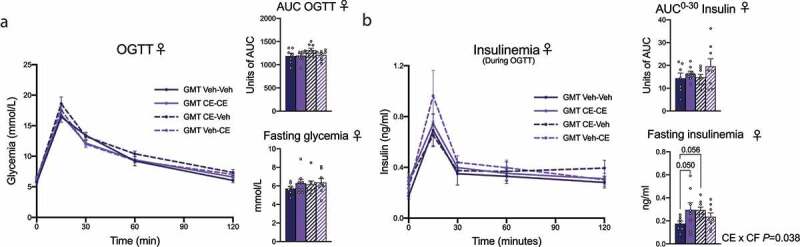
Effect of GMT on glucose homeostasis in young female offspring. (a) Female offspring were fasted for 12 h and an OGTT was performed. (b) Insulinemia during the OGTT was measured by ELISA. Two-way ANOVA with a Holm–Sidak *post hoc* test was used to assess the significance of the differences between groups. Two-way ANOVA with repeated measures with a Holm–Sidak *post hoc* test was used to assess differences between groups at different time points. Data are expressed as the mean ± SEM (n = 7–8/group)

To establish the causal role of the gut microbiota in the observed metabolic changes in female offspring, we next performed GMT in 4-week-old female mice depleted of microbiota following antibiotic treatment (Figure 9a). Initial body weight was similar between treated groups after randomization (Figure 9b). When assessing body weight gain 5 weeks post-GMT, we did not observe significant changes between treated group of mice (Figure 9c). However, GMT Veh-CE treated mice had the highest body weight gain. Food intake was similar between groups (Figure 9d). Consistent with higher body weight gain, we observed that several white fat depots were increased in GMT Veh-CE-treated mice as compared to GMT CE-Veh and GMT CE-CE-treated groups, which was statistically significant in mesenteric fat (mWAT) mass (Figure 9e). The weight of the brown adipose tissue (BAT) was also significantly increased in the GMT Veh-CE-treated group compared to the GMT Veh-CE-treated mice and significantly reduced in GMT CE-Veh group compared to GMT Veh-Veh-treated group (Figure 9e). The weight of the soleus and gastrocnemius was not different between treated groups (figure 9f). We next assessed body composition and did not observe any difference in fat and lean masses (Figure 9g). Liver weight and triglycerides were similar between GMT-treated groups (Figure 9h). Four weeks post-GMT treatments, we performed an OGTT and observed similar fasting glycemia and glucose tolerance between groups (Figure 10a). Insulinemia during the OGTT did not reveal any significant difference between treated groups, although the peak insulin response at 15 min tended to be higher in GMT Veh-CE mice (Figure 10b). Calculation of the AUC^0-30^ min confirmed that GMT Veh-CE and to a lesser extent GMT CE-CE mice tended to have an exacerbated insulin response. We observed an interaction between both factors on fasting insulinemia, which suggests an exacerbation of the phenotype associated with an HFHS diet in non-fostered offspring nursed by a CE-dam (Figure 10b). Our results are in accordance with the general metabolic phenotype reported in HFHS-fed Veh-CE female offspring and suggest that at least part of the altered metabolic phenotype of these mice is linked to postnatal alterations in the gut microbiota environment.

## Discussion

The purpose of this study was to determine the contribution of the obesogenic *in utero* vs. postnatal environments in the development of future metabolic outcomes and the establishment of the gut microbiota in offspring using the cross-fostering technique. The administration of a polyphenol-rich CE to diet-induced obese dams during the preconception, gestation and lactation periods was used as a strategy to induce metabolic and microbial alterations. The CE polyphenol treatment in dams was associated with an improved metabolic phenotype as shown by their increased insulin sensitivity, reduced hepatic fat and adipose depots and a shift in the global gut microbiota community structure. However, administration of the polyphenol-rich CE to nursing dams exacerbated the development of metabolic outcomes associated with obesity in female offspring, but not in male offspring, an effect that was linked to changes in the gut microbiota. The establishment of the gut microbiota in female offspring was found to partly underlie the predictive value of the postnatal environment on the development of metabolic outcomes in offspring.

Our group previously demonstrated that CE treatment can prevent and even reverse diet-induced metabolic disorders in obese male mice and that these effects were associated with important gut microbiota modulations.^21,22^ However, to our knowledge, this study is the first to demonstrate the beneficial effects of the administration of a polyphenolic-rich CE in the context of obesity in dams during pre-pregnancy, the gestation and lactation periods. Another animal study has also observed decreased liver weight and improved insulin sensitivity in dams having consumed a grape seed procyanidins extract (GSPE),^33^ whereas others have shown no effect.^34,35^ However, these studies were conducted in dams that have been fed a standard diet during the gestation and/or lactation periods. Therefore, more studies are needed to confirm our results in dams and to assess the effect of other sources of polyphenols more specifically in the context of obesity during distinct periods such as preconception, gestation or lactation. Furthermore, the role of the gut microbiota has not been explored in these studies. The implication of the gut microbiota in driving these metabolic improvements is of high interest considering the growing literature documenting the important role of polyphenols, particularly proanthocyanidins from cranberry polyphenols, in shifting the gut microbiota composition to promote host benefits.^20,36–38^ In our study, CE-dams significantly clustered apart from Veh-dams on the PCoA plot. Moreover, the overexpression of *Muribaculaceae_g* observed in CE-dams, previously known as S24-7,^39^ is in accordance with more recent studies that have demonstrated a positive association between the increased relative abundance of this bacterium and a lean phenotype.^40,41^ Lately, Cao *et al*.^42^ found *Muribaculaceae* family to be increased in mice resistant to HFD-induced obesity. Until recently, this family was vastly ignored despite its dominance in the gut microbiota of rodents.^39^ It is also worth noting that we have recently reported an increased abundance of *Muribaculaceae_g* in diet-induced obese mice supplemented with a proanthocyanin-rich blueberry extract for 12 weeks,^37^ in line with the potential role of these bacteria in the beneficial effects of polyphenols.

Most studies evaluating the effects of polyphenols in dams during the gestation and/or lactation periods on obesity-associated disorders in offspring focused on male offspring only,^43–46^ even if it is well known that the development of metabolic homeostasis is the subject of important sexual differences.^47^ Accordingly, we show herein that the administration of a polyphenol-rich CE during the nursing period worsens the development of obesity-associated comorbidities in female offspring but not in male offspring. Indeed, Veh-CE and CE-CE female offspring had increased final body weight, adipose tissue depots and lean mass. Del bas *et al*.^48^ showed that the administration of a GSPE to dams fed an HFD during pregnancy and lactation increased the weight of different white adipose tissues in offspring compared to their counterparts whose dams did not receive the GSPE. However, those changes were associated with a reduced plasma inflammatory profile in the epididymal adipose tissue compared to their HFD-fed counterparts that were not exposed to the GPSE and were only assessed in male offspring. Glucose tolerance was also impaired in Veh-CE and CE-CE female offspring. Similar results have already been reported, but again, only in male offspring. Mice fed a chow or a cafeteria (CAF) diet born from dams receiving an STD diet and supplemented with GSPE were more insulin-resistant and had lower insulin sensitivity compared to their respective counterparts born from dams receiving the vehicle.^33^ The same group also demonstrated that male offspring born from a dam fed an STD and receiving the GSPE were more prone to cardiovascular diseases.^34^

Most importantly, the design of our study allowed us to distinguish the effect of the polyphenol-rich CE administered to dams between the prenatal and postnatal environments. The postnatal environment had a stronger impact than the prenatal environment on the future metabolic outcomes and the associated changes in the gut microbiota in male and female offspring. The strong influence of the postnatal environment has been previously suggested^16,19^ but remains debatable among the scientific community. The microbiome acts as an important vector of transmission between dams and offspring during this period to such an extent that it has been estimated that only 2–9% of the gut microbiome is determined by heritability and that environmental factors are mostly shaping the gut microbiome.^49^ Many authors suggest that there is a critical «window of opportunity» for the establishment of the gut microbiota and immune development in both mice^50^ and humans,^51^ from which the consequences of a disruption during this phase can be seen over the entire lifespan. Therefore, the acquisition of a distinct gut microbiota profile by male and female offspring nursed by a CE-dam at T0 could have contributed to the exacerbated metabolic phenotype seen only in female offspring. Indeed, transfer of female offspring ceacal content into young microbiota-depleted female mice for 5 weeks was associated with increased weight of some adipose tissues in GMT Veh-CE-treated mice as opposed to CE-Veh-treated mice, as well as a strong trend to increase glucose-stimulated insulin secretion, which was observed only 4 weeks post-GMT. In preterm infants, females tended to have higher α-diversity than males after the first 10 d of life, an effect that reached significance after 30 d of life.^52^ Although we did not observe sex differences in our mouse model when specifically assessing α-diversity (data not shown), we saw important β-diversity variation between groups in female offspring at T0, which were only seen in male offspring at T8. The most remarkable finding is the similar impact that Veh-dams had on the establishment of the gut microbiota in both male and female offspring during the postnatal period, but not in male and female offspring nursed by a CE-dam. *Peptococcaceae*_*g*, which was found to be overrepresented in CE-CE and Veh-CE female offspring but not in male offspring, has been associated with murine obesity,^53^ intestinal inflammation^54^ and anxiety-like behaviors.^55^ Interestingly, Bridgewater *et al*.^55^ have also observed an increased in *Peptococcaceae_g* that was only detected in HF-fed female but not male mice. This could therefore explain why male offspring, being less susceptible to the acquisition of this bacterial genus, were less metabolically affected by the acquisition of an unfavorable microbiota.

GMT experiments performed in young female mice only partly reproduced the altered phenotype of CE-treated female offspring. It is possible that a longer period of time is needed for the colonized microbes to promote all of the phenotypic changes observed in the female offspring. However, it is also likely that other complementary environmental factors contribute to influence metabolic outcomes in offspring during the postnatal period. First and foremost, the cross-fostering itself has to some extent contributed to modulate the metabolic phenotype of female offspring nursed by a CE-dam, an effect that was more strongly observed in the GMT experiments; notably on insulinemia. Moreover, lactation is a vector of transmission taking place between dams and offspring during the postnatal stage of development and considered as an important source of bioactive substances. Milk contains small but non-negligible amounts of bacteria (10^3^–10^4^ CFU/ml) that can colonize the gut microbiota of the infant.^56^ Gut microbiota-derived polyphenol metabolites have also been found in the milk of lactating mothers.^57^ In our study, we have not collected milk samples during the lactation period of dams. Hence, we are not able to rule out some role of breast milk in determining the long-term metabolic phenotypes in female offspring. It should also be noted that Hsieh *et al*.^58^ reported that reducing 40% of milk triglycerides in mammary gland adipocytes and white adipose tissue using a phosphoenolpyruvate carboxykinase^−/−^ mouse model contributed to the development of insulin resistance in offspring. It is therefore possible that the reduced fat mass observed in dams receiving the CE could have contributed to triggering unwanted metabolic disorders in female offspring by reducing milk triglycerides. Finally, we cannot exclude that epigenetic effects of breast milk components could also have contributed to the phenotypic effects seen in offspring. Indeed, developmental epigenetic modifications are not only restricted to the intrauterine environment but also occur throughout early life, particularly in rodents as the gestation period is short and offspring are born immature.^59^

## Conclusion

In summary, our study shows for the first time the beneficial health properties associated with cranberry polyphenol consumption in dams on obesity-associated metabolic disorders during the pre-pregnancy, pregnancy and lactation period. Our data also revealed the occurrence of a sex-specific metabolic exacerbation of the weight gain and glucose intolerance in offspring associated with the consumption of polyphenols by dams, pointing toward the importance of the postnatal environment in shaping future metabolic outcomes. It is further suggested that the acquisition of a distinct gut microbiota in female offspring nursed by a CE-dam in the postnatal environment compared to male offspring partly contributes to the observed deleterious phenotype, but that complementary environmental factors also likely exert a significant influence. These findings certainly warrant further investigation to understand the contribution of the different postnatal modulators (i.e.microbial-derived polyphenolic metabolites, lactation, epigenetic modifications) in mediating future and sex-dependent metabolic alterations in offspring. The finding of such strong sexual dimorphism further highlights the importance of studying both sexes when assessing dietary outcomes in animal models.

## Supplementary Material

Supplemental Material

## Data Availability

The data that support the findings of this study are available from the corresponding author, AM, upon request.
