# Supplementary material for: The postnatal window is critical for the development of sex-specific metabolic and gut microbiota outcomes in offspring
Source: Gut Microbes. 2021 Nov 23;13(1):2004070. doi: 10.1080/19490976.2021.2004070 (PMC8632343; doi:10.1080/19490976.2021.2004070)
Supplement: Supplemental Material [file KGMI_A_2004070_SM5418.zip › Supplementary information/Supplementary material captions.docx]

**Supplementary Table 1. Characterization of the polyphenol-rich CE extract.**

Supplementary Table 2. Male offspring biological parameters. The effect of the cross-fostering on offspring’s biological parameters is referred to as “CF”. The effect of the treatment of the nursing mother (CE vs Veh-dams) on offspring’s biological parameters is referred to as “Tx”. Two-way ANOVA analysis with a Holm-Sidak *post hoc* test was used to evaluate the difference between groups. Retroperitoneal adipose tissue (rpWAT); Epidydimal adipose tissue (eWAT); Mesenteric adipose tissue (mWAT); inguinal white adipose tissue (iWAT); Brown adipose tissue (BAT); Visceral adipose tissue (VAT). Data are expressed as the mean ± SEM (Veh-Veh n=16; CE-CE n= 8; CE-Veh n=17; Veh-CE n=15).

**Supplementary Table 3. Female offspring biological parameters.** The effect of the cross-fostering on offspring’s biological parameters is referred to as “CF”. The effect of the treatment of the nursing mother (CE vs Veh-dams) on offspring’s biological parameters is referred to as “Tx”. Two-way ANOVA analysis with a Holm-Sidak *post hoc* test was used to evaluate the difference between groups. rpWAT: retroperitoneal adipose tissue; oWAT: ovarien adipose tissue; mWAT: mesenteric adipose tissue; iWAT: inguinal white adipose tissue; BAT: brown adipose tissue; VAT: visceral adipose tissue. Data are expressed as the mean ± SEM (Veh-Veh n=17; CE-CE n=9; CE-Veh n=9; Veh-CE n=7).

**Supplementary Figure 1. Contribution of the postnatal environment to the development of the gut microbiota in offspring.** β-diversity of the fecal microbiome at T0 was observed between postnatal groups in male and female offspring using means of PCoA on Bray-Curtis dissimilarity index (n=12/group). (A) Male-Veh vs Male-CE; (B) Female-Veh vs Female-CE; (C) Male-Veh vs Female-Veh; D) Male-CE vs Female-CE. Ellipses on the PCoA represents 95% confidence interval. Bray-Curtis distance between dams (biological or adoptive) and fostered male and female offspring at (E) T0 and (F) T8. Differences in distances between biological or adoptive dams and offspring were assessed using a paired t-test for parametric data set and Wilcoxon rank test for non-parametric data set. Data are expressed as the mean ± SEM. * *P*<0.05.
