# Supplementary material for: The postnatal window is critical for the development of sex-specific metabolic and gut microbiota outcomes in offspring
Source: Gut Microbes. 2021 Nov 23;13(1):2004070. doi: 10.1080/19490976.2021.2004070 (PMC8632343; doi:10.1080/19490976.2021.2004070)

Supplementary Table 1

Polyphenols type	Polyphenols content
**Total polyphenolic compounds** (g/100g (%) dry weight – eq. Gallic acid)	34,2±2,9
**Proanthocyanidins** (mg/100g dry weight – eq. Epicatechin)	10697,3
Monomers	739,8
Dimers	2440,4
Trimers	1556,1
Tetramers	971,3
Pentamers	791,6
Hexamers	24,6
Heptamers	99,2
Octamers	94
Nonamers	69,3
Decamers	0
Polymers >10	3910,9

Monomers, dimers and trimers of proanthocyanidin (DP 1–3). Oligomeric and polymeric proanthocyanidins (DP >3). DP, degre of polymerisation.

Supplementary Table 2. Male offspring metabolic phenotype

	Veh-Veh	CE-CE	CE-Veh	Veh-CE	Two-way ANOVA
**Male offspring metabolic phenotype**					
**Initial body weight (g)**	11,70±0,60	11,20±0,30	10,90±0,60	11,30±0,30	—
**Final body weight (g)**	28,60±0,60	29,80±0,70	28,60±1,10	29,60±0,60	—
**Body weight gain (g)**	16,90±0,80	18,70±0,70	17,70±0,70	18,30±0,50	—
**rpWAT (g)**	0,34±0,03	0,45±0,06	0,34±0,06	0,38±0,04	—
**eWAT (g)**	1,15±0,07	1,44±0,18	1,31±0,18	1,28±0,12	—
**mWAT (g)**	0,26±0,02	0,37±0,05	0,30±0,03	0,31±0,024	—
**iWAT (g)**	0,38±0,02	0,48±0,04	0,42±0,042	0,44±0,032	—
**BAT (g)**	0,09±0,01	0,13±0,02	0,11±0,01	0,11±0,01	—
**VAT (g)**	1,75±0,11	2,25±0,28	1,99±0,27	1,95±0,19	—
**Soleus muscle (g)**	0,017±0,00	0,017±0,00	0,018±0,00	0,017±0,00	—
**Gastrocnemius muscle (g)**	0,24±0,01	0,26±0,004	0,25±0,01	0,25±0,01	—
**Fat mass (g)**	3,90±0,26	4,50±0,56	4,10±0,50	4,00±0,37	—
**Fat mass gain (g)**	2,78±0,26	3,59±0,54	3,16±0,45	3,14±0,35	—
**Lean mass (g)**	19,60±0,30	20,30±0,28	19,60±0,46	20,10±0,30	—
**Lean mass gain (g)**	9,53±,87	10,75±,65	10,16±,37	10,44±0,62	—
**Total food intake (kcal)**	690,2±15,86	707,0±17,71	701,4±16,51	726,1±11,72	—
**Liver weight (g)**	0,980±0,034	0,990±0,05	0,954±0,04	0,986±0,03	—
**Hepatic triglycerides (ug/mg tissue)**	19,36±1,79	24,61±4,50	19,22±2,10	21,70±1,70	—

Supplementary Table 3. Female offspring metabolic phenotype

	Veh-Veh	CE-CE	CE-Veh	Veh-CE	Two-way ANOVA
**Female offspring metabolic phenotype**					
**Initial body weight (g)**	10,70±0,50	11,60±0,20	10,20±0,40	11,0±0,20	—
**Final body weight (g)**	22,90±0,50	23,70±0,50	22,10±0,40	23,80±0,80	**Tx**
**Body weight gain (g)**	12,20±0,50	12,10±0,40	11,90±0,60	12,80±0,70	—
**rpWAT (g)**	0,26±0,04	0,37±0,07	0,18±0,05	0,22±0,06	**CF**
**oWAT (g)**	0,48±0,07	0,59±0,08	0,39±0,04	0,62±0,14	**Tx**
**mWAT (g)**	0,21±0,03	0,23±0,04	0,18±0,01	0,25±0,04	—
**iWAT (g)**	0,28±0,025	0,34±0,027	0,24±0,01	0,31±0,05	**Tx**
**BAT (g)**	0,07±0,01	0,08±0,01	0,06±0,01	0,07±0,01	**Tx**
**VAT (g)**	0,94±0,13	1,18±0,18	0,74±0,08	1,08±0,23	**Tx**
**Soleus muscle (g)**	0,015±0,00	0,015±0,00	0,014±0,00	0,015±0,00	—
**Gastrocnemius muscle (g)**	0,21±0,004	0,22±0,00	0,20±0,01	0,22±0,01	**Tx**
**Fat mass (g)**	2,20±0,21	2,60±0,30	1,80±0,15	2,50±0,49	—
**Fat mass gain (g)**	1,17±0,21	1,47±0,27	0,83±0,17	1,45±0,49	—
**Lean mass (g)**	16,30±0,23	16,70±0,25	16,0±0,21	16,60±0,30	**Tx**
**Lean mass gain (g)**	7,64±0,45	6,87±0,37	7,83±0,39	7,60±0,39	
**Total food intake (kcal)**	674,9±13,31	725,4±28,51	667,6±13,58	730,9±31,59	**Tx**
**Liver weight (g)**	0,86±0,02	0,88±0,05	0,77±0,03	0,80±0,06	**CF**
**Hepatic triglycerides (ug/mg tissue)**	21,25±1,50	25,55±3,10	21,48±2,80	22,20±2,10	—

Supplementary Figure 1

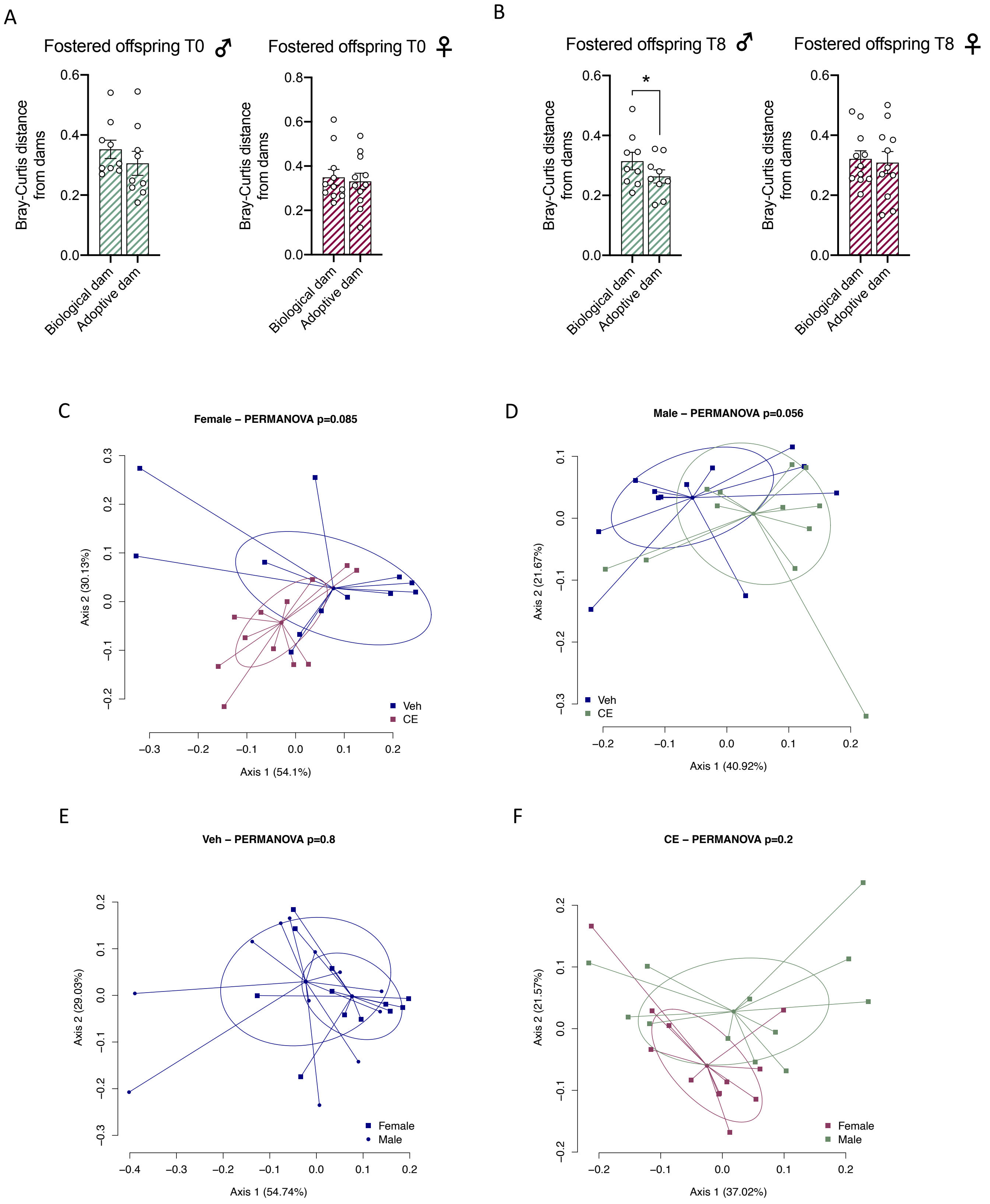

Supplement: Supplemental Material [file KGMI_A_2004070_SM5418.zip › Supplementary information/Supplementary material.pdf]
